# Single cell genomics based insights into the impact of cell-type specific microbial internalization on disease severity

**DOI:** 10.3389/fimmu.2024.1401320

**Published:** 2024-05-21

**Authors:** Jyoti Soni, Rajesh Pandey

**Affiliations:** ^1^Division of Immunology and Infectious Disease Biology, INtegrative GENomics of HOst PathogEn (INGEN-HOPE) Laboratory, Council of Scientific & Industrial Research-Institute of Genomics and Integrative Biology (CSIR-IGIB), Delhi, India; ^2^Academy of Scientific and Innovative Research (AcSIR), Ghaziabad, India

**Keywords:** intracellular microbes, immunogenomics, host-microbe interaction, infectious disease, single-cell RNA-seq

## Abstract

Host-microbe interactions are complex and ever-changing, especially during infections, which can significantly impact human physiology in both health and disease by influencing metabolic and immune functions. Infections caused by pathogens such as bacteria, viruses, fungi, and parasites are the leading cause of global mortality. Microbes have evolved various immune evasion strategies to survive within their hosts, which presents a multifaceted challenge for detection. Intracellular microbes, in particular, target specific cell types for survival and replication and are influenced by factors such as functional roles, nutrient availability, immune evasion, and replication opportunities. Identifying intracellular microbes can be difficult because of the limitations of traditional culture-based methods. However, advancements in integrated host microbiome single-cell genomics and transcriptomics provide a promising basis for personalized treatment strategies. Understanding host-microbiota interactions at the cellular level may elucidate disease mechanisms and microbial pathogenesis, leading to targeted therapies. This article focuses on how intracellular microbes reside in specific cell types, modulating functions through persistence strategies to evade host immunity and prolong colonization. An improved understanding of the persistent intracellular microbe-induced differential disease outcomes can enhance diagnostics, therapeutics, and preventive measures.

## Highlights

- Genomics technologies like Single-cell RNA sequencing offer insights into intracellular microbe interactions within specific cell types, aiding in combating infectious diseases effectively.
- It provides insights into the potential functional role of yet unculturable microbes for the wholistic understanding of the factors towards differential disease severity, although infected by similar or same pathogen/s.
- The dynamic interplay between hosts and microbes impacts human health and disease via molecular signals and microbial communities.
- Microorganisms serve beneficial roles like aiding digestion or can cause diseases and infections.
- Intracellular pathogens, including bacteria and viruses, pose significant health risks by evading immune responses and causing persistent infections.

## Introduction

The dynamic interaction between a host and its resident microbes, including bacteria, fungi, and viruses, is a complex, dynamic, and multifaceted process that influences various aspects of host health (1) (2). This interaction is distinguished by molecular signals, biochemical communication, and a nuanced equilibrium of microbial communities, all of which have substantial implications for our well-being (3) (4). While some microorganisms are beneficial, playing crucial roles in processes such as digestion and immune system priming, others with pathogenic properties can lead to diseases and infections (5) (6). Previous studies, such as the Human Microbiome Project (HMP), have provided insights into the human microbiome in terms of human health and diseases. The first phase of this project successfully revealed the microbial communities of the human body, while the second phase, Integrative HMP (iHMP), uncovered host–microbiome interactions using the “omics” approach. The findings from these studies highlight that microbial composition and diversity vary among individuals (7).

Post-COVID-19, there has been renewed interest in whether and to what extent the presence of microbes would impact clinical severity and disease outcome after infection with a primary pathogen, like SARS-CoV-2, Dengue or *Mycobacterium tuberculosis*. Infectious diseases pose a persistent threat to human health because pathogens exploit host–microbe interactions to initiate infections and cause severe diseases. Understanding the complex interplay between hosts and microbes is crucial for developing effective countermeasures against these diseases. The impact of infectious diseases caused by microorganisms extends beyond posing a global burden on public health systems (8). An outstanding example is the recent outbreak caused by SARS-CoV-2, which has caused severe mortality worldwide (9). Infectious diseases, including COVID-19, dengue, and tuberculosis, are the leading causes of death worldwide, especially in low and middle-income countries (LMICs). To alleviate the burden of infectious diseases and enhance global public health, it is essential to examine each type of microbe separately to gain a better understanding of the problem (10).

Based on their immunopathology, these microbes can be classified as intracellular or extracellular. Although extracellular bacterial infections are generally easier to treat, intracellular bacteria pose significant threats to human health. Certain microbes can infiltrate host cells to harness cellular resources and conceal themselves from host defenses (11). Other microorganisms, such as *Salmonella enterica* and *Listeria monocytogenes*, can adapt their lifestyle to become intracellular for a limited period. These microorganisms use diverse endocytic mechanisms to enter and evade phagocytic and non-phagocytic cells, thereby avoiding host defense mechanisms. Identification of intracellular microbes is complex because of the limitations of traditional culture-based methods, which hinder timely and accurate identification and characterization. Despite these constraints, there is growing recognition of the significant impact of these microorganisms on host cells. Therefore, it is essential to understand the intracellular diversity of these microorganisms and their impact on health and disease. This article emphasizes the genomics approach in understanding the interaction between host and intracellular microorganisms. These microorganisms may preferentially infect specific cell types for their survival and replication, and this preference may be influenced by factors such as functional responsibilities, availability of nutrients, immune evasion mechanisms, and replication opportunities (12). These elements collectively contribute to the microorganisms’ capacity to flourish within the host cell. Various cell types can host or carry microorganisms, including epithelial cells, immune cells (13), endothelial cells, and fibroblasts (14). The diverse array of cell types contributes to differential immune responses and varying susceptibility to infection by various microorganisms (15). Several studies have indicated that HIV selectively targets CD4^+^ T cells, *Mycobacterium tuberculosis* and *Chlamydia pneumoniae* primarily affect macrophages, Epstein–Barr virus targets B cells (16), and hepatitis B virus primarily targets hepatocytes (17) (18). Although the role of immune cells in protecting the body from foreign invaders is widely acknowledged, certain microorganisms have evolved to thrive within these cells (19). Once these microbes infiltrate the host cells, they can manipulate the host’s immune response by evading phagocytosis, the complement system, and innate immune receptors (20). In addition, they hinder apoptosis, exhibit resistance to host effector mechanisms and trigger immune dysfunction, i.e., immunosuppression.

While previously viewed as potential contamination, microbial RNA inadvertently captured in host transcriptome datasets is now recognized in a limited way for its potential to elucidate diverse yet dynamic host-microbiome interactions. A study by Bost et al. allowed simultaneous profiling of infected host cells and taxonomic profiling of the responsible viruses using the Viral-Track approach (21). This method has successfully differentiated hepatitis B-infected cells in human clinical samples and identified specific host factors associated with the viral replication. The study was done on bronchoalveolar lavage (BAL) samples from the COVID-19 patients. Similarly, Lloréns-Rico et al. used an in-house pipeline for the analysis of single-cell RNA sequencing (scRNA-seq) datasets derived from the bronchoalveolar lavage (BAL) samples collected from both COVID-19 patients and non-COVID-19 pneumonia controls. Their analysis revealed a specific bacterial subset associated with various host immune cell populations, such as neutrophils, monocytes, and macrophages (22).

However, it is of utmost importance to consider whether the presence of intracellular microbes’ influences disease severity. To address this, we need a comprehensive understanding of the dynamics of microorganisms specific to various cell types and their functional significance. Conventional methodologies are limited to only known culturable microbes because of their inherent fastidiousness and the time required to adapt according to the culture conditions. In a different approach, bulk RNA sequencing involves studying the combined genetic material of millions of cells, which provides a profile of the average gene functioning across a large group of cells. These methods restrict our ability to understand individual cells and intracellular microbes. Nevertheless, scrutiny of intracellular microorganisms that are specific to certain cell types can be achieved using other evolving methodologies that can identify intracellular microorganisms and their genomic constituents. Single-cell RNA sequencing is a classic example of such a technique (23). Single-cell RNA sequencing (scRNA-seq) allows the unbiased evaluation of cellular heterogeneity by providing comprehensive genome-wide molecular profiles across a diverse range of individual cells (24). This innovation in genomic research has made it possible to sequence intracellular microbial genomes, as a by-product of single cell whole transcriptome sequencing (WTA), thereby facilitating a deeper understanding of their interactions with specific host cells, and enabling the development of effective strategies to combat various infectious diseases (25) (26). In this perspective article, we discuss the role of intracellular microbes within specific cell types and their potential influence on the outcomes of infectious diseases. It also emphasizes emerging technologies that enhance our understanding while addressing associated challenges.

## The burden of infectious diseases

### Bacteria, viruses, and co-infections

**Figure 1:**
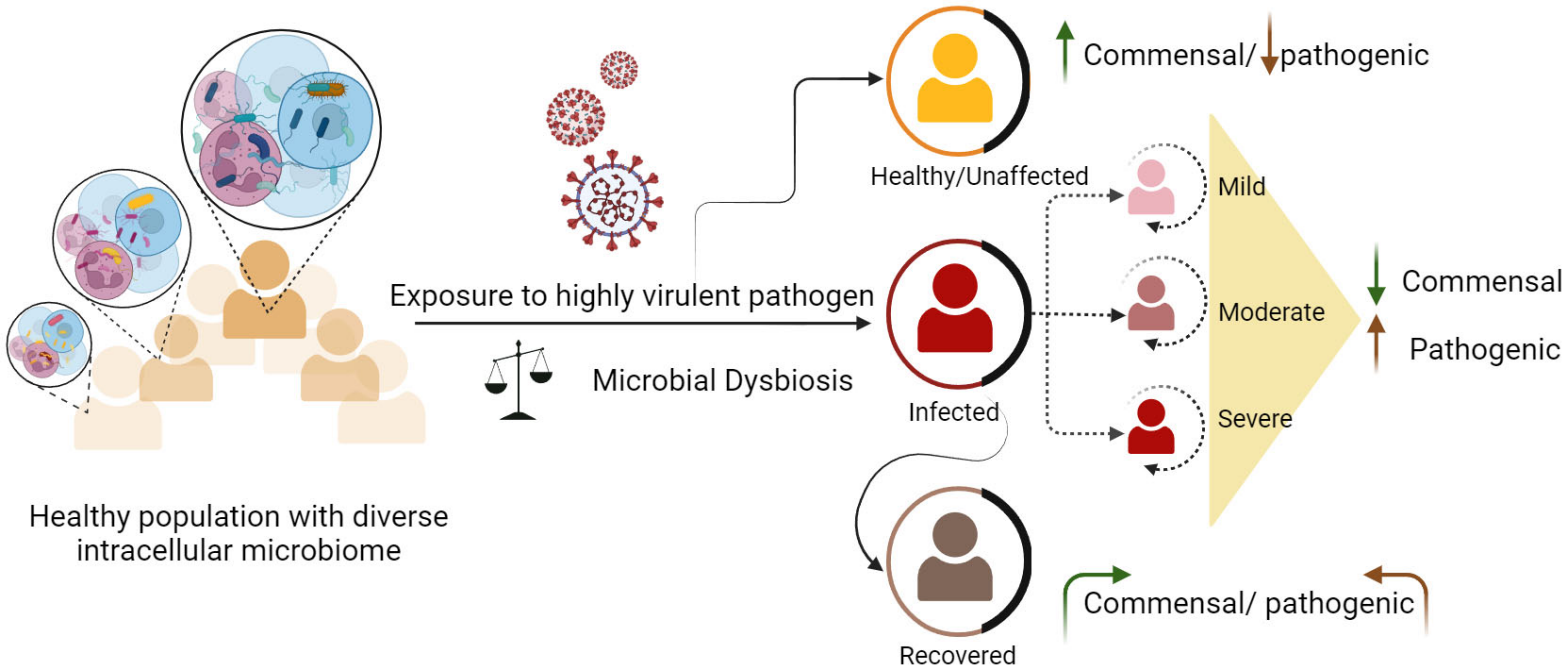
Dynamics of intracellular microbes and differential disease outcomes among healthy individuals. Healthy individuals carry diverse and heterogeneous microbial species. Exposure to any harmful pathogen can lead to microbial dysbiosis. Disruptions in existing microbial communities can either exacerbate the severity of certain diseases in some individuals or augment their ability to combat the infection, resulting in a milder or asymptomatic outcome.

According to the World Health Organization (WHO), infectious diseases rank as the second most prominent cause of global mortality (27). Despite considerable advancements in the realm of antibiotics and vaccines, significant challenges persist globally, where diseases such as COVID-19, dengue, malaria, tuberculosis, and HIV continue to impose a significant burden. Pathogens that cause infectious diseases include bacteria, viruses, fungi, and parasites (28). Within this microbial milieu, some microbes endure substantially for longer periods inside the host and can cause persistent infections (29). The outcome of these infections can vary, either symptomatic or asymptomatic, depending on factors such as host immunity, microbial load, strain variations, genetic predispositions of the host, existing health conditions, age, gender, pathogen type, and environmental factors (30) (31). In community outbreaks of infectious diseases, different disease clinical phenotypes, ranging from mild to severe, lead to various consequences of recovery from the disease or mortality (32) (**Figure 1**). To understand infectious diseases caused by a specific pathogen, we mainly focus on the interaction between the host and the pathogen, neglecting the dynamics of microbial components within the cells (33). The ultimate response of the host results from complex interactions orchestrated by various immune components (34). The cells responsible for these responses contain dynamic microbial components that may regulate their functional characteristics. The presence of intracellular microbiota influences the interactions between various cell types and their subsets. In a study by Derek et. al., it was elucidated that certain epithelial cells exhibit elevated levels of gene expression related to host defense against microbes, and glycosylation, whereas others exhibit a more focused pattern of gene expression for pattern recognition receptors (35). This shows that the complex interplay between the host and microbe, which impacts metabolism and other vital biological pathways, significantly shape disease outcomes. The involvement of the gut microbiome in modulating host physiology and compositional change in bacterial infections such as Salmonellosis, tuberculosis, and viral infections such as influenza, hepatitis, SARS CoV-2, has already undergone extensive study (36) (37). This influence extends to a range of other diseases, including tumorigenesis and infectious diseases such as malaria and chickenpox. The intricate mechanisms employed by these microbes to manipulate various host processes remain unclear. A thorough understanding of the specific cell types targeted by different microbes is necessary to elucidate host-microbe dynamics.

### Global impact of intracellular bacterial infections

Recent findings reveal that 7.7 million deaths worldwide have been attributed to bacterial infections. Only *Staphylococcus aureus, Escherichia coli, Streptococcus pneumoniae, Klebsiella pneumoniae*, and *Pseudomonas aeruginosa* account for >50% of deaths globally (38). Depending on their lifecycle, these bacteria can be extracellular or intracellular. By adopting an intracellular life cycle, these bacteria easily escape from the phagocytic cells, complement system, and antibodies. Once internalized, they find a suitable niche either in the cytosol or in other organelles. Although, unlike viruses, bacteria can replicate on their own, for protection and nutritional needs, some choose to thrive inside the host cell niches. However, immune cells can recognize these microbes via various antigens or pathogen-associated molecular patterns and destroy infected host cells. Intracellular bacteria use dynamic mechanisms to survive within host cells and avoid the host’s external immune defenses (39) (40). Inside the host, these bacteria infect a range of cell types. The major reservoirs of these bacteria include immune cells such as monocytes/macrophages and dendritic cells (DCs), along with B and T cells (41) (42). Furthermore, non-professional phagocytes such as epithelial cells, fibroblasts, and endothelial cells also serve as reservoirs for these bacteria. It’s important to note that not all intracellular bacteria cause disease; some simply ensure their survival within host cells in symbiosis (**Table 1**).

**Table 1:** Intracellular bacteria and their mechanisms of evading the host's defense mechanisms.

S. No	Microbe(s)	Cell type(s)	Evasion mechanisms	Disease(s)	Ref.
1.	*Mycobacterium tuberculosis*	Macrophages, epithelial cells, endothelial cells, dendritic cells, and neutrophils	• Inhibition of phagolysosome formation, via tryptophan-aspartate containing coat protein • Hiding in lipid droplets • Autophagy inhibition • Increase the production of proinflammatory cytokines, TNF-α, IFN-γ, IL-8, and IL-1β	Tuberculosis, pulmonary complications, autoimmune diseases, and metabolic syndromes	(43–45)
2.	*Salmonella typhi*	Macrophages, dendritic cells, neutrophils, and B cells	• Inhibition of NF-κB signalling • Inhibits dendritic cell migration • Inhibition of autophagy	Typhoid fever	(20, 46)
3.	*Listeria monocytogenes*	Macrophage, epithelial cells, endothelial cells, hepatocytes, and dendritic cells	• Escape into the cytosol (listeriolysin O mediates) • Immunomodulation by various virulence factors	Listeriosis and meningeal or systemic infections	(47, 48)
4.	*Brucella abortus*	Macrophages, dendritic cells (DC), trophoblastic cells (TE), neutrophils, B cells	• Prevents the fusion of autophagolysosomes • Inhibits activation of NK cells and maturation of DCs • Inhibits complement system and antigen presentation	High fever and, brucellosis	(48–50)
5.	*Shigella flexneri*	Epithelial cells and macrophages,	• Manipulate host innate immune responses (T3SS effector mechanism) • Induces apoptosis prevention of NF-κB activation	Bacillary dysentery(shigellosis)	(48, 51, 52)
6.	*Staphylococcus aureus*	Neutrophils, macrophages, and B cells	• Resist phagocytic oxidative and nitrosative killing. • Trigger apoptosis/necroptosis	Bloodstream infections, pneumonia, and endocarditis	(38, 48)
7.	*Anaplasma phagocytophilum*	Neutrophils, eosinophils Macrophage, DCs, endothelial cells	• Remodelling of the cytoskeleton. • Inhibition of cell apoptosis, and • Manipulation of the immune response	Granulocytic anaplasmosis	(51, 52)
8.	*Legionella pneumophila*	Macrophages, monocytes, and epithelial cells	• Abrogation of phagosome-lysosome fusion • Antigenic variation • Induces apoptosis by activating caspase-3	Legionellosis	(11, 53)
9.	*Chlamydophila pneumoniae*	Epithelial cells, monocytes, macrophages, dendritic cells (DCs), lymphocytes, and neutrophils.	• Suppresses the production of reactive oxygen species and nitric oxide • Inhibits apoptosis for longer persistence	Bronchitis, pharyngitis, sinusitis, pneumonia	(11, 41)
10.	*Rickettsia rickettsii*	Endothelial cells, monocytes, macrophages, and hepatocytes	• Induce apoptosis • Inhibition of infection-induced activation of NF-κB	Rocky Mountain spotted fever	(11, 54)

Host and bacteria co-evolved over a long period, during which some bacteria modified their ability to survive inside the host cells and adapt to the host defense systems. If such co-operation does not develop between the host and microbes, it might result in the eradication of one of the parties involved (55). In addition, there are other repercussions, such as partial digestion, inactivation of bacteria, and inhibition of cell death processes within infected cells. Throughout this interaction between the host cell and bacteria, there is an increased frequency of protein and metabolite transportation, as well as direct gene transfer (54). A bacterium that has undergone lysis releases its RNA into the cellular microenvironment (56). Consequently, cells harboring intracellular microorganisms tend to possess DNA/RNA originating from the bacterial species. If we envisage the relative proportion of human host/bacterial RNA from the holo-transcriptome perspective, the quantity of RNA from bacteria (non-replicative) may be relatively low compared to the host cells transcriptome, yet it can be effectively captured by mapping back the unmapped reads to microbial genomes (57).

Although numerous intracellular bacteria have been extensively studied from the perspective of microbiology, these studies have not emphasized the evolving field of immuno-genomics, which is crucial for gaining a deeper understanding of the role of microbial entities (especially non-culturable ones) present within a single cell. It is necessary to understand the functional roles, pathogenicity, mutations, and other factors (to be discovered) that can be harmful. While bulk RNA-Seq studies provide valuable insights into the host-pathogen interaction of *Mycobacterium tuberculosis* (Mtb) with macrophages, they fail to reveal the cellular heterogeneity within the macrophage cell population. Gene expression is differentially regulated in various cells, which possess unique and specific roles. The field of transcriptomics has witnessed significant advancements in the application of single-cell sequencing, primarily because of its enhanced resolution capabilities. A single-cell-based study by Huang et al., characterized two main macrophage populations: alveolar and interstitial macrophages, indicating that alveolar macrophages, with their anti-inflammatory M2-type characteristics, create an environment favorable for *Mycobacterium tuberculosis* (Mtb) replication and dissemination. In contrast, interstitial macrophages, which are associated with a more stressful immune milieu, pose challenges to Mtb (58). For example, the growth characteristics of *Mycobacterium tuberculosis* exhibit substantial variation among individual cells, particularly in terms of growth, cell cycle duration, and cell size (43) Nathan et al. (59) employed scRNA-seq to focus on memory T cells isolated from peripheral blood mononuclear cells (PBMC) of TB-progressing patients. The differentiation of memory T cell states revealed distinct clusters, with the TH17 subset showing differential abundance and function between progressors and non-progressors. Another single-cell study by Reuter et al. provided valuable information on the functionality of SPI2-T3SS, the formation of Salmonella-induced filaments (SIF), and the intracellular proliferation of different serovars in diverse host cell types. In addition, a genomic approach can help identify microbes that are in an inactive state as these microbes can become active in future favorable environments. Such insights can be effectively utilized to formulate groundbreaking and specific methodologies for the management and prevention of infectious diseases that arise due to intracellular microorganisms.

## Intracellular viral dynamics: understanding the impact on the host

### Decoding viral intracellular mechanisms during the SARS-CoV-2 pandemic

Viruses, on the other hand, are obligatory parasites much smaller than bacteria that can exploit and use host cells for replication and proliferation. The identification and quantification of viral genomes is important for understanding viral diversity and evolution. Viruses predominate microbial infections because they rapidly evolve to new variants. The challenge in the identification and characterization of these viruses which can often lead to a new disease (21). The recent global outbreak caused by SARS-CoV-2 highlighted the intricate interplay between viruses and host cells at the genomic level. The physiological consequences of SARS-CoV-2 involve a dual mechanism: direct *invasion* of host cells and modulation of intercellular signaling (60). This dual mechanism contributes to the complexity of the virus’s impact on the host, encompassing both the direct damage caused by infection and the broader systemic response triggered by the immune system. The pandemic was associated with an increasing risk of viral mutations that were particularly advantageous and resulted in new variants of concern (VOC). Numerous studies have shown that multiple SARS-CoV-2 VOCs (Alpha, Beta, Delta, and Omicron) elicit diverse cellular responses in heterogeneous tissues and cell types. Chattopadhyay et al. reported the downregulation of CD22-CD45 interactions, which is responsible for B cell maturation and aberrant T cell activation via the dysregulation of CD40-CD40LG signaling in the COVID-19 positive individuals (61). Another study by Wendao et al. showed the downregulation of MHC class II molecules in B cell types (62). Robinson et al. showed the existence of SARS-CoV-2 in macrophages, neutrophils, and, lymphocytes using single-cell transcriptomic data, and they also showed the change in immune response genes in cells containing viral reads as compared with bystander cell types (63).

### Patterns of dengue outbreaks

Dengue virus (DENV) constitutes a significant menace to public health in tropical countries, with approximately 40% of the global population facing the risk of dengue infection (64). At present, dengue fever has affected over 100 nations situated in the tropical and subtropical regions (65) (66). Dengue virus has four serotypes with envelope and spherical particles. The genome of each serotype encodes ten proteins, including structural (M, E, C) and non-structural (NS1-NS5) components. Internalization relies on membrane fusion, endocytosis, and pH-dependent fusion, facilitated by M and E proteins. Dengue can be severe, with or without any warning signs (decreased platelet count, liver enlargement, vomiting, mucosal bleeding and so on). Some studies have indicated that the presence of other microorganisms, particularly those capable of inducing co-infection, in conjunction with dengue virus is associated with complications such as pneumonia and extended fever, along with an increased probability of mortality (65). This research evidence highlights the need for further investigation into the possible relationship between the pathogenesis of dengue virus and pre-existing intracellular microorganisms or secondary infections. The research question may focus on uncovering changes in the composition of intracellular microorganisms during dengue infection and whether any specific species had an impact on influencing the disease phenotype toward recovery or mortality. Exploring these aspects would offer valuable insights into the mechanisms of viral infections and the various phenotypes that they can result in.

### Dynamic transitions: viral life cycles and their consequences

Other viruses such as herpesvirus, papillomavirus, adenovirus, and polyomavirus are considered commensal viruses in the human body (67). When a virus successfully enters a host cell and starts making multiple copies of its genetic material, it causes lytic infection. Successful execution of the viral lytic cycle requires the viral genome to integrate into host cell chromosomes. In the latent phase, viral genomes remain in a stable (quiescent) state within the host cell, not actively replicating but poised for future activity (68). The ability of latent infections to transition back into a lytic state plays a crucial role in the spread of viruses from infected to uninfected individuals. In their inactive or slowly growing state, these viruses remain a significant concern because they can still make healthy individuals sick (69). Even in healthy individuals, we carry viral components within our cells (70) (71). Certain viruses, such as retroviruses, possess the capability to invade cells and incorporate their genetic material into the host genome. Consequently, these viral genetic elements become a permanent component of the host cell and can be passed down to subsequent generations of cells (72). For instance, HIV infiltrates the nucleus and forms a complex for integration, allowing its DNA to merge with the genetic material of the host cell (73). This indicates how the virus genome significantly impacts its host in different ways, apart from causing typical viral diseases. The investigation of the viral component present within our cells concerning health and diseases necessitates further scrutiny to fully comprehend its implications. Adenovirus, dengue virus, Ebstein–Barr virus, human cytomegalovirus, human papillomavirus, poliovirus, hepatitis C virus, human immunodeficiency virus, and Vaccinia virus are known to activate various metabolic processes (glycolysis, pentose phosphate pathway etc.) that are required for intracellular replication and proliferation (74). Because we often inherit systemic viruses from our parents early in life, the virome also plays a role in our genetic identity (75). These viral elements that reside in our chromosomes can govern gene activation, influence genetic changes, and transcribe specific genes for crucial protein production. Endogenous retroviral-derived regulatory elements have been implicated in the regulation of immune cell development, activation, and response to pathogens (76). However, the immune system is continually engaged, interacting with the body’s microbiome, including the virome, even in the absence of immediate threats (77). This interaction shapes the host’s unique immune profile, which can differ among individuals and change over time (78). Traditional techniques for viral studies have many limitations, including the fact that not all viruses can be cultured in the laboratory. Using conventional sequencing and molecular approaches, viral genomes can now be rapidly captured and tracked for their evolution. These techniques are required for the rapid detection of new or re-emerging viruses that impact human health and development (79).

### Intracellular fungal infections

Globally, fungal infections are exceedingly common and can result in severe infections, varying from asymptomatic or mild cases to potentially life-threatening systemic infections. The epidemiology of fungal diseases has witnessed significant changes in recent decades. Notably, *Aspergillus, Candida*, and *Cryptococcus* species are the major fungal pathogens responsible for most serious fungal diseases. Some fungi, including *Candida albicans*, are naturally present in our microflora and help protect us from harmful pathogens (80) (81). However, these normally harmless species can become harmful, particularly in immunocompromised individuals. In addition, certain fungi can survive inside cells and manipulate the host’s cellular functions to ensure their survival. However, some pathogenic fungal species can cause severe infections, which are often associated with high rates of illness and death. The understanding of diverse intracellular fungal species in infections and diseases is still incomplete, urging further research to dissect these essential temporal sequences.

## Co-Infection chronicles- the leading cause of worse disease outcomes

Because of our constant exposure to various pathogens, a substantial portion of the population unknowingly harbors chronic or latent infections, including those caused by viruses, bacteria, or parasites. The microbiota comprises many potential pathogenic species. Therefore, it is highly probable that any new infection will manifest as a co-infection (82). The traditional view of a single host and single pathogen interacting with one another has been challenged by numerous studies, that have introduced the concept of co-infections. Co-infection can occur either due to the reactivation of pre-existing pathogenic microorganisms in the presence of a new pathogen or because of the co-colonization of the host by new pathogenic species. Co-infection modulating the outcome of infections is now emerging as an important area in understanding multiple co-existing pathogens and the host response (83). Some examples of fungal co-infections with primary viral and bacterial infections includes-

- Influenza viruses and Aspergillus infections can cause severe pneumonia by damaging the lungs and impairing their function. Immune response to these infections can also contribute to lung injury, leading to ARDS and respiratory failure (84).
- COVID-19-associated pulmonary aspergillosis was reported to reduce the number of CD4^+^ and CD8^+^ T cells leading to immune disruption in COVID-19 patients. High inflammation and direct damage to the airway epithelium during COVID-19 enabled Aspergillus pathogenesis.
- Mucormycosis has been reported to occur in COVID-19 patients who receive systemic corticosteroid treatment or have diabetes. The increased presence of glucose-regulated proteins, which act as receptors for fungal attachment and invasion, increases the likelihood of co-infection (85).
- Aspergillus and *Pseudomonas aeruginosa*: Patients with cystic fibrosis (CF) may be affected by both *Aspergillus fumigatus* and *Pseudomonas aeruginosa*, and their interaction can lead to worsening of lung function and clinical outcomes.
- *Mycobacterium tuberculosis*, and cryptococcosis are opportunistic pathogens that can cause morbidity and mortality by evading the host immune system and residing in phagocytes.

The co-presence of microbes in a particular habitat leads to various interactions between them and the infecting primary pathogen. This co-presence results in reciprocal interactions between host-pathogen, host-microbes, and microbes-pathogen, which may result in co-infection (44). However, many of these microbes remain dormant for a long time, but possibly upon infection with any harmful pathogen changes the cellular homeostasis (dysbiosis), which may result in their reactivation. Because of the difficulties in the identification of dormant microbes, co-infection at the cellular level and its consequences in human health and infectious diseases require needs more attention.

## The implications of co-infection modulation on therapeutic strategies and patient management

Effective management of co-infections requires a comprehensive approach that includes early screening to identify and select specific antimicrobial treatments. Co-infections often resemble other common infections, which can complicate diagnosis and create uncertainty regarding the necessity of antibiotics. Crucial measures included ensuring environmental cleanliness, practicing thorough hand hygiene, and implementing screening and isolation protocols. Antimicrobial stewardship is vital to reduce the occurrence of drug-resistant infections. Prolonged hospital stay, illness, inadequate surveillance, and excessive antibiotic use can contribute to the development of co-infections. Following patient safety guidelines, bundles can reduce the risk of coinfection. Overall, an integrated multimodal strategy is necessary to manage co-infections (86).

## Application of genomics to understanding cell-type-specific intracellular microbes

Genomics involves the identification of genes and functional components in the genome of an organism. It is an invaluable tool for understanding the impact of intracellular microbes within specific cell types, including their involvement in cellular dysfunction, immune response, and disease development. Recently, Yadav et al. reported the presence of dynamic intracellular microbial species within peripheral blood mononuclear cells (PBMCs) in healthy, infected and recovered individuals from SARS-CoV-2 using scRNA-seq (12). Furthermore, unique tools such as PathogenTrack and Yeskit have been developed to identify intracellular pathogens from scRNA-seq datasets, allowing for the assessment of transcriptomic characteristics at the individual cell level. In addition, a genomics-driven approach has indicated that most of major human cancer types possess an intra-tumoral microbiota. Direct interactions between these microbial communities and immune cells were investigated using co-culture techniques.

The presence of a microbial genome can be a consequence of the following, i) the intracellular presence of dormant or inactive forms of microbes, which may remain within host cells without actively replicating or causing harm, ii) microbial remnants persist even after successful elimination by the host’s defense mechanisms, including their genetic material (DNA or RNA), which then undergoes lysis within the cells (87), iii) uptake of microbial products, (RNA, proteins, and metabolites) from the microbes residing in the extracellular environment by certain cell types, iv) uptake of apoptotic bodies generated from infected cells, and v) transport of exosomes or vesicles containing microbial RNA, from infected cells to neighboring cells, which may unhouse microbes.

## Challenges and pitfalls in intracellular microbe research

Recent research has greatly enhanced our understanding of the human microbiota including, skin, oral, gut and organ-specific microbes. Nevertheless, many unknowns and challenges remain in disentangling intracellular microbes and differential infection outcomes.

### Cellular hibernation: insights into dormancy

Most microorganisms in the natural world live in frequently unpredictable environments that subject them to stress and challenges that are not optimal for their growth and reproduction (88). Despite difficult conditions, such as extreme temperatures or limited resources, microbes have the remarkable ability to temporarily slow down various biological processes. This reduction in metabolic activity and increased resistance to heat, antibiotics, and various stressors enables them to conserve energy and resources by effectively halting their vital life processes until conditions improve (89). They can save energy and resources because of the decrease in metabolic activity, which effectively suspends their essential life processes until the situation improves. The ability of these organisms to tolerate stressful conditions and resume normal activities when the environment stabilizes through dormancy eventually increases their chances of survival and reproduction. During any acute infection or disease, a given treatment with antibiotics may negatively affect active microbes, in which the subpopulation of dormant microbes remains unaffected, persists for longer, and may become active in the future. Microbiology mainly focuses on studying microbes that grow quickly because of methodological constraints. Emerging technologies can provide a better granular and genomic-based understanding of dormant and resuscitated intracellular microbes and their components (45).

### Transcriptionally active microbes

Once the microbes have favorable conditions, they become active in transcribe the genes required for their proliferation and pathogenicity. Numerous host genes, especially immune genes, are activated as the cells encounter any microbe. The human genome comprises of approximately 20,000 genes, that are regulated in a tissue-specific manner. The expression of these genes can be altered by both internal and external signals. However, the microbial genome can be considered our “second genome”. The interaction between the host and pathogenic microbes can induce cellular and immune dysfunction and cause infectious diseases. With the advent of advanced technology, we can now meticulously assess the genome sequences of various microbes with increased accuracy. Hence, for microorganisms to effectively establish a specialized environment within the cellular structure and for the host cells to eliminate the microbe, a conflict arises between the two entities. The essential element in this process is the dynamic interplay between the genetic material of the host cell and the microbial genes. Transcriptionally active microbes, possess the capability to modify the functional properties of host cells, and engage in intricate interactions with the cellular machinery of the host organism. Identification of transcriptionally active microbes requires the capture of RNA profiles representing active life processes and interactions between host and microbes.

## Conflict, compromise, and cooperation between microbes and host immunity

**Figure 2:**
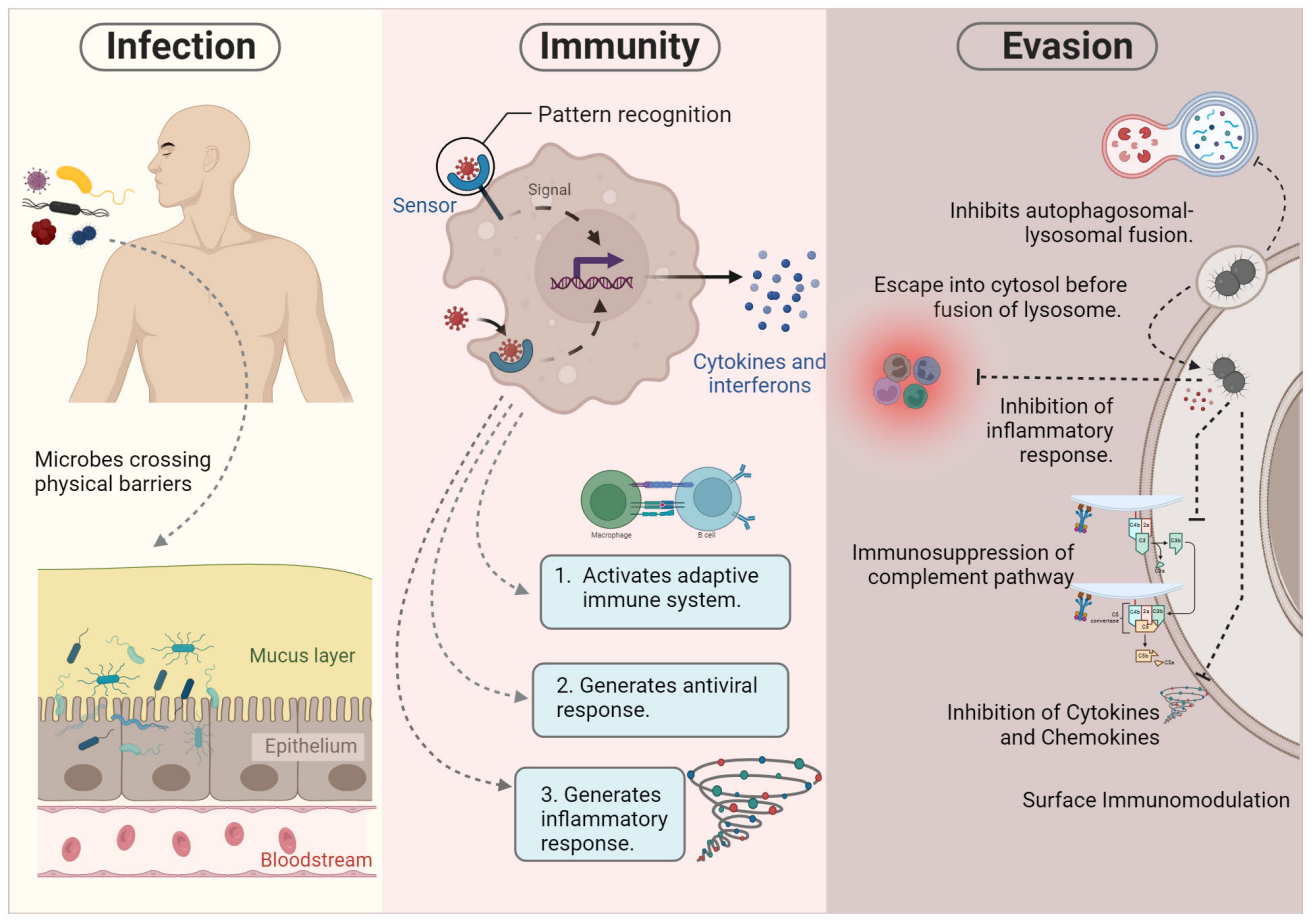
Pathogenesis of intracellular microbes. During infection, certain microbes bypass physical barriers such as the skin and mucous epithelia. This initial breach triggers the activation of the innate immune response. Successful execution of innate immunity can lead to either the elimination of the microbe or the initiation of the adaptive immune system for further processing. However, microbial survival strategies may hinder killing mechanisms, allowing evasion of the immune system. These evasion tactics include inhibiting autophagolysosome fusion, residing in specialized vacuoles, escaping into the cytosol, and impeding various immune components such as inflammatory responses, complement pathways, cytokines, and chemokines.

The innate immune system comprises a range of defense mechanisms within the host, including physical and chemical barriers. It uses a wide array of mechanisms to combat a broad spectrum of microbial threats and remove harmful substances, including toxins and allergens. Although the innate and adaptive immune systems are often depicted as separate, they typically work in tandem. The innate immune system relies on several components, including complement proteins, phagocytic cells (such as monocytes, macrophages, and neutrophils), and natural killer (NK) cells, to provide immediate defense against various threats. These host defense mechanisms are inaccessible to intracellular microbes. The components of the innate immune system, such as antigen-presenting cells, further activate the adaptive immune system, which employs a more powerful immune response to eliminate infectious agents. In addition, certain microbes have developed the capability to persist within the host despite the presence of both innate and adaptive immune responses. For example, pathogens such as *M. tuberculosis, S. enterica*, and *Neisseria* can evade clearance by the immune system even after continuous immune surveillance (90) (**Figure 2**).

## Innate immunity against intracellular microbes

The interaction between intracellular microbes and the host follows a cascade of immune reactions in which the host activates various microbicidal mechanisms at different stages of infection, while the microbes employ different evasion strategies. In particular, for intracellular microbes, both phagocytic and non-phagocytic cells are involved in microbial uptake and target intracellular destruction. Understanding the immune response involved in intracellular killing can deepen our understanding of cell-type-specific mechanisms and the pathogenesis of infection. The first counteraction against intracellular microbes is provided by the components of the innate immune system. The products released by intracellular bacteria are recognized by Toll-like receptors (TLRs) and Nod-like receptors (NLRs), and activates the effector phagocytic cells (46). Phagocytic cells generally recognize microbes based on unique molecular patterns (PAMPs) in the microorganisms, irrespective of their pathogenicity (11). TLRs are responsible for initiating a response that prompts macrophages to produce proteins and peptides with antimicrobial properties. They also activate the transcription of genes involved in the production of reactive oxygen species (ROS), which permits lysosome fusion (91). Some pathogenic microbes, such *as Leishmania, Staphylococci, Coxiella* and *Salmonella*, are well organized to survive even the acidic environment and continue to replicate. For example, PhoP/PhoQ regulation in *Salmonella* helps its intracellular survival (34). Together, these pathogen recognition receptors (PRRs) result in the production of antimicrobial peptides and induction of the inflammatory response mediated by interferons and pro-inflammatory cytokines (TNF-α). Macrophages can also be activated via natural killer cells through interferon (IFN-γ) production, which plays a major role in the killing of intracellular pathogens (92).

### Cytokines and chemokines as a host defense against intracellular microbes

The activation of the host innate immune response results in the production of diverse effector molecules, such as cytokines, chemokines, and other microbicidal proteins. These molecules are released by certain cell types in response to damage or recognition of any foreign invader inside the body. Pro-inflammatory cytokines are produced predominantly by the NK cells, activated or infected macrophages, and monocytes. IFN-γ is one of the most important cytokines against intracellular infections, along with TNF-α, it activates macrophages to kill intracellular microbes. Certain studies have also suggested the function of cytokines including IFN-γ, type I IFNs, IL-1, IL-6, IL-15, and IL-18, in the activation of other immune cells responsible for microbial infections (47). Importantly, the microbial nature and stimulated cell type define the cytokine pattern released by cells (34). These cytokines act on their receptor molecules to generate effector functions in specific cell types. However, intracellular microbes can inhibit the production of these molecules by releasing proteins that mimic their receptors, such as IL-2 in S. typhimurium, TNF-α release in *Yersinia enterocolitica*, and *Legionella pneumophila*, which inhibit IL-2 (53).

Chemotactic cytokines, which activate and provide a source for the migration of various immune cells, predominantly leukocytes, are known as chemokines. Chemokines are critically induced by early detection of intracellular microbes, to recruit diverse array of immune cells that are specialized for microbial killing, such as professional antigen-presenting cells (APC). Chemokines are further categorized into different families, in which CC chemokines target macrophages, monocytes, dendritic cells, T cells, and NK cells. The CXC group is mainly involved in neutrophil chemotaxis.

### Humoral and cell-mediated immunity to different intracellular microbes

The adaptive immune response is typically activated by the innate immune response in the defense of the host. Unlike the body’s inherent defenses, adaptive immunity, is remarkably capable of recognizing certain antigens and initiating a focused defense against infections. Humoral immunity against intracellular microbes is mediated by B-cell activation. Some bacteria can infect and replicate inside B cells, including *Salmonella thyphimurium, Brucella, Mycobacterium tuberculosis, Listeria monocytogenes, Francisella tularensis*, and *Helicobater* spp. Antigens presented by the major histocompatibility complex are further recognized by T cells, which differentiate into their subset cells for effector functioning. CD4^+^ mediates the adaptive immune response against intracellular microbes. The subsets of T cells respond to antigen presentation, and downstream, they activate killer cells to eradicate the pathogen. In contrast to innate immunity, which is species-specific, adaptive immunity differs among individuals within a species in response to specific antigenic challenges. Its diversity leads to an extremely adaptable spectrum of functions, which allows it to identify millions of different antigenic compounds. A critical characteristic of the adaptive response is the generation of long-lived memory cells that remain in a quiescent state but can rapidly regain their defensive functions upon encountering the specific antigen again. This immunological memory empowers the adaptive response to significantly enhance host defenses when facing the same pathogens or toxins on subsequent encounters, even after an extended period, providing an efficient and robust protective response. The adaptive immune response ultimately results in antigen-specific activation of the effector mechanisms of the innate immune system.

## Subversion of host defense by intracellular microbes

### “It requires maximum exertion to maintain a stationary position” - Lewis Carroll

Certain microbes have evolved in tandem with their host in response to each other, effectively neutralizing any cellular defense mechanisms. Intracellular microbes are capable of thriving within immune and non-immune cells. This evolutionary arms race between hosts and pathogens is driven by genetic differences that determine how well the host can recognize and respond to microbial components, and how the pathogen can evade or overcome the host’s defenses. Entry inside cells provides additional benefits to the bacteria. Immune cells provide a nutritious and safe niche for these microorganisms (48). While entering a host, numerous microbes evolved multiple anti-immune mechanisms to evade and modulate the host immune response to ensure its survival. Microbes thwart host defenses by using various tactics such as modulation of microbial surfaces, secretion of immune-modulators, antigenic variation, inhibiting lysosomal fusion (93), altering lysosomal pH for cytosolic escape (94), using protease-like activating factors for host protein degradation and neutrophil deactivation, hindering humoral immunity and inflammatory responses, blocking antigen processing and presentation (61), inducing immunosuppression via the complement regulatory pathway, suppressing reactive oxygen species and nitric oxide production, and inhibiting apoptosis, ensuring prolonged persistence within the host. Collectively, these strategies allow microbes to navigate and evade different components of the host immune system, ensuring their sustained survival and persistence.

## Technology’s frontiers: recognizing and addressing limitations

**Figure 3:**
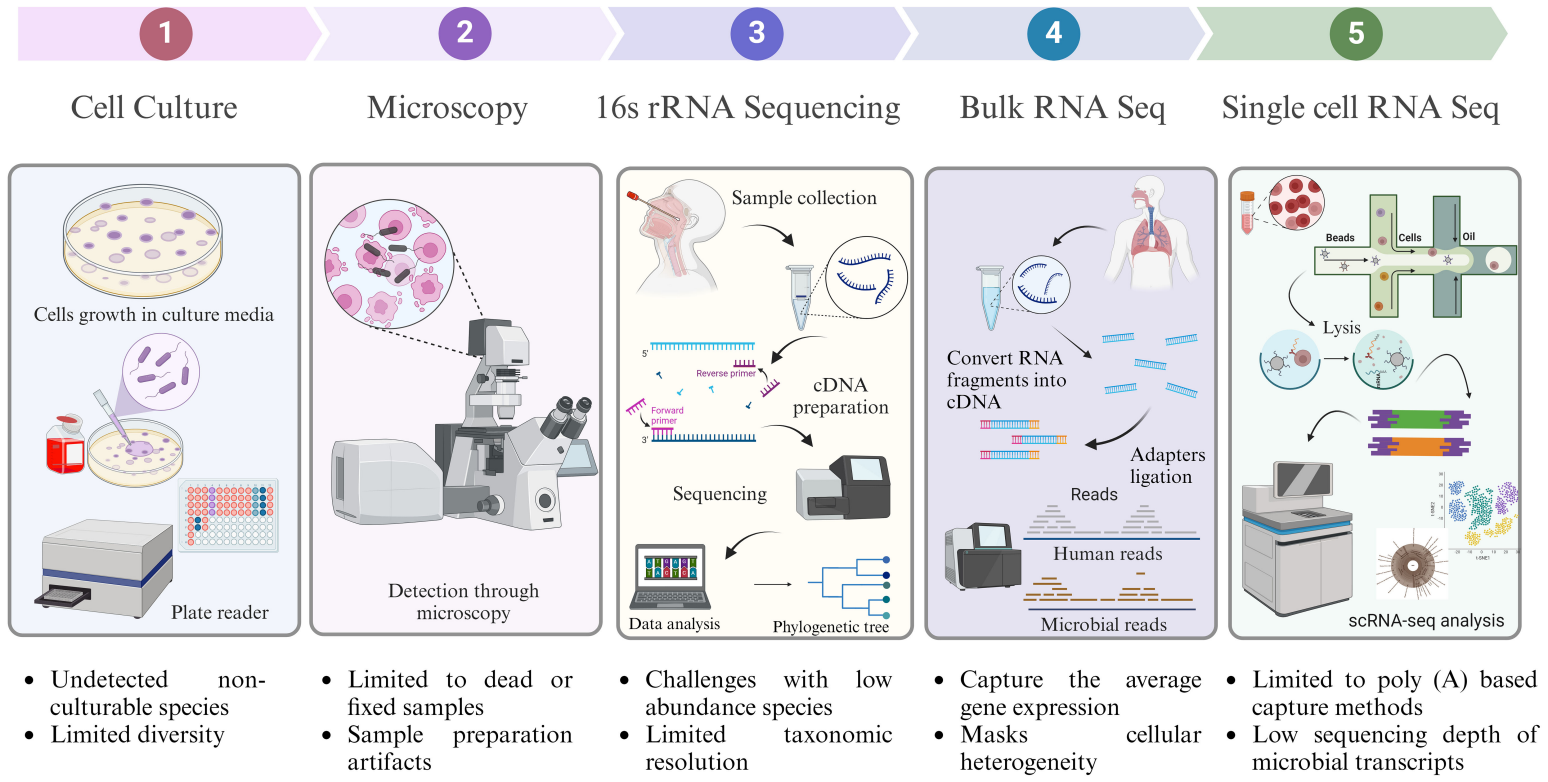
Traditional and advanced techniques for studying intracellular microbes. The exploration of intracellular microbes encompasses both conventional and cutting-edge methodologies. Conventional approaches, such as culture-based and microscopy-based techniques, have inherent limitations, particularly concerning the diversity and comprehension of non-cultivable microorganisms. In contrast, advanced techniques, notably next-generation sequencing technology, transcend the constraints of traditional methods. The most sophisticated among them is single-cell analysis, which offers unparalleled resolution down to the individual cell and strain levels.

Despite decades of research, we still have limited insights into the factors that regulate the severity of infection between individuals. Studying intracellular microbes, especially through culture-based methods, fails to detect scrupulous or metabolically active but unculturable bacteria. One of the challenges in accurately describing the current state of microbial diversity is the difficulties associated with environmental circumstances (pH, osmotic pressure, nutritional components) (95). The major limitations of traditional techniques include the following: i) these methods are time-consuming, as they require the isolation and cultivation of microorganisms in the laboratory; ii) the diversity and complexity of microbial communities may not be accurately known, and iii) not all microorganisms can be cultured, and challenges such as environmental conditions, stress, or acclimatization can hinder their recovery (**Figure 3**).

Microscopy allows the direct visualization of intracellular microbes, providing valuable morphological information. It is limited by the resolution and size of the microbes that can be visualized, whereas NGS can detect and identify microbes at the molecular level, even if they are too small to be seen under a microscope. Microscopy allows studying a small number of microbes at a time, making it difficult to capture the full diversity and complexity of microbial communities. Conventional microscopy-based methods for identifying intracellular bacteria also have drawbacks, such as resolution limitations that make distinguishing between different species difficult. Precisely localizing internal microorganisms may require additional labelling techniques.

Culture-independent methods, such as metagenomics, metatranscriptomics, metaproteomics, and metabolomics, provide insights into the functional potential and activities of microbial communities in their natural environment (96). Metagenomics involves studying the genomes of host and microbial organisms. Metatranscriptomics provides information on total gene expression in a specific environment. Metaproteomics and metabolomics are other omics branches based on protein expression and metabolite identification and quantification, which help us to understand the active microbial pathways (97) (98).

## Single-cell transcriptomics: revolutionizing the exploration of microbial diversity

One might wonder what motivates the in-depth investigation of these microorganisms at the single-cell level. It is important to determine the functional role of intracellular microbes inside specific cells and whether they can modulate the severity of diseases. The answer lies in the fact that various tissues, each responsible for unique physiological functions, exhibit diversity and specialized functions attributed to variations in gene expression and unique characteristics among their constituent cells. Individual cells within tissues show heterogeneity in gene expression and function. A high-throughput cutting-edge alternative emerged, single-cell technology, which can address these limitations, as it enables the sequencing of individual cells and provides different information, including proteomic, epigenomic, transcriptomic, and metagenomic, thereby offering a more comprehensive view of cellular diversity and function.

Bulk RNA sequencing revealed microbes in different samples, including blood (99). However, various cell types, make it challenging to study the specific interactions between microbes and individual cell types such as neutrophils (100). Because of their limited lifespan and inability to be cryopreserved, they are difficult to study. Our understanding of distinct cell and tissue types is compromised because heterogeneity among these cells remains masked. Furthermore, there is still a significant gap in our understanding of which microbes are associated with specific immune cells. This knowledge is particularly vital in the context of immune cells, which play a pivotal role in combating infections and pathogens. It is essential to understand the composition of pre-existing microbes and how they evolve throughout the progression of diseases (101). The key to our knowledge of human infection biology is how the immune system combines signals and coordinated responses from many cell types and how inter-individual diversity in these cell types translates to variations in infection outcomes.

**Figure 4:**
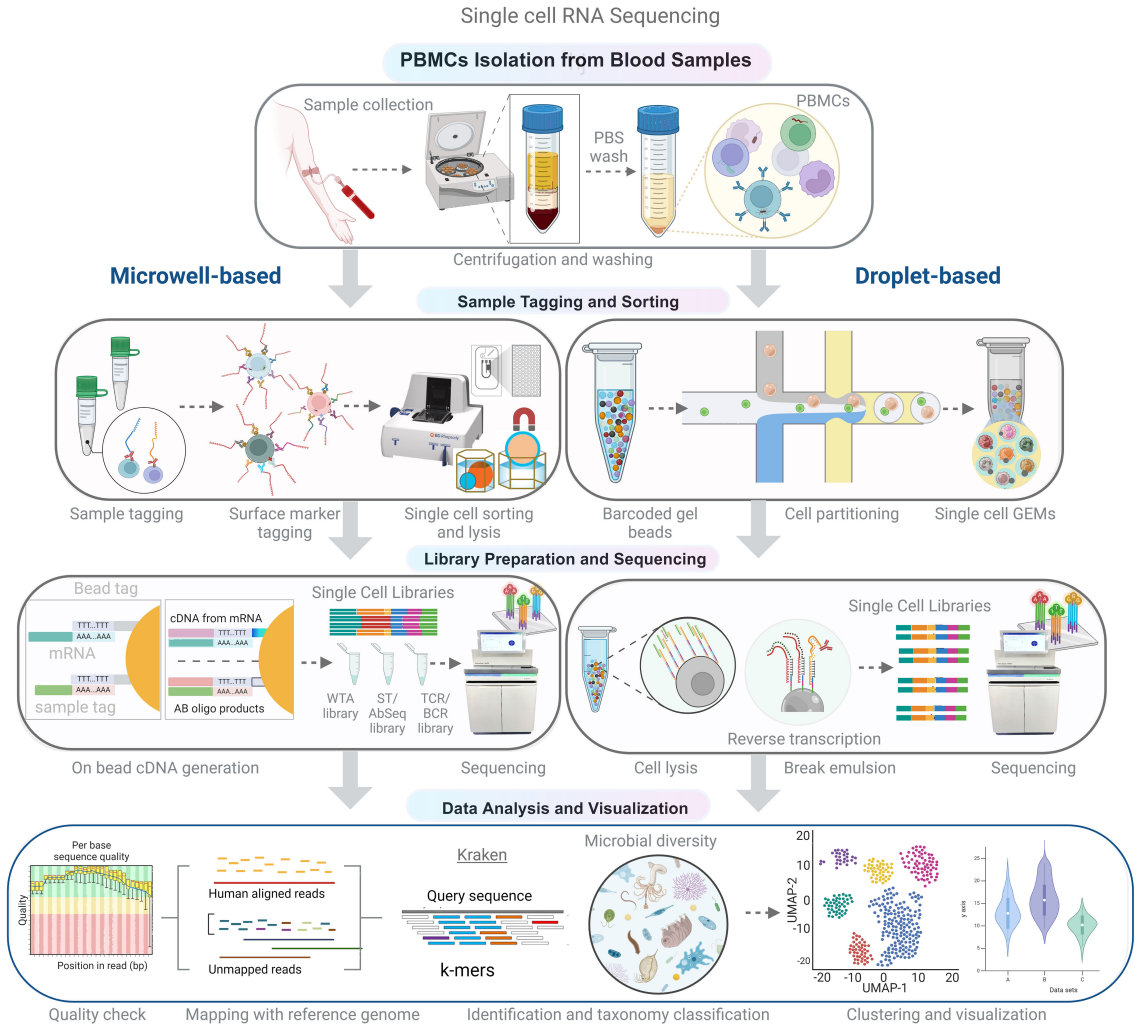
Schematic representation of the experimental and analytical workflow for single-cell RNA sequencing (scRNA-seq). Peripheral blood mononuclear cells (PBMCs) are isolated from whole blood using density gradient centrifugation. Single-cell suspensions are then used for library preparation, which can be achieved using microwell or droplet-based methods to capture single cells and barcoded beads. Following cDNA synthesis and amplification, the libraries are sequenced using high-throughput sequencing platforms. In the analytical phase, non-human reads are extracted and analyzed to identify microbial sequences, allowing for the comprehensive characterization of both host and microbial transcriptomes in a single-cell resolution.

Single-cell RNA-seq (scRNA-seq) technology has made recent advancements that enable the disintegration of complex tissues and host compartments into cell types and their importance in health and diseases. A scRNA-seq study by Hoffman et al. revealed the diverse outcomes of bacterial infection by macrophage subsets. Some cells may allow the replication of microbes, some may inhibit the growth and eliminate the microbe, while some may probably not respond to either side (49). The primary steps involved in scRNA-seq techniques are isolation and capture of single cells, cell lysis, reverse transcription, cDNA amplification, and library preparation. However, these processes vary in procedure depending on the sequencing platform. Among these steps, the isolation and sorting of individual cells are critical yet labor-intensive processes within the single-cell RNA-seq (**Figure 4**). Cell separation techniques encompass various methods, such as fluorescence-activated cell sorting (FACS), magnetic-activated cell sorting (MACS), microfluidics (10x Genomics), and microwell-based systems (BD Rhapsody), which are geared toward high-throughput applications. The design of the microwell system is particularly noteworthy because it allows the accommodation of individual cells coupled with a single bead. Subsequently, the process involves cell lysis and mRNA hybridization to the beads, followed by reverse transcription and amplification for sequencing, which serves as a universal PCR priming site. It is equipped with a combination of elements, including a distinctive cell label, a unique molecular index, and an mRNA capture sequence composed of deoxythymidine/oligo (dT). To date, single cell RNA-seq data have mainly been used to investigate host response by analyzing mRNA expression. Accordingly, analyzing either host or microbe alone may limit the understanding of host-microbe interactions. Although, simultaneous study of host and microbe genomes increases the complexity, scRNA-seq can elucidate the influence of diverse intracellular microbial species on the composition and host transcriptional profiles of diverse cells. The “dual RNA-seq” studies allow us to investigate both host and pathogen genomes in parallel (11) (102). New state-of-the-art tools have been developed to analyze the single cell transcriptomic datasets to identify intracellular pathogens at the single-cell level (50, 51). Hence, it can capture all transcripts, including microbial/bacterial transcripts having poly A (not necessarily more than 50 A) (103). Undoubtedly, not all microbes exhibit poly-A tails in their transcripts; nevertheless, a significant majority of microbes manifest this trait, thereby accentuating the value of this approach in elucidating bacterial diversity.

Single-cell transcriptomics has ushered in formidable methodologies for determining microbial diversity. Pioneering-modified techniques, such as Fluidigm C1, Prokaryotic single-cell RNA sequencing (PETRI-seq), microbial split-pool ligation transcriptomics (microSPLiT), multiple annealing and dC-tailing-based quantitative single-cell RNA-seq (MATQ-seq), and Bacterial droplet-based single-cell RNA-seq (BacDrop), have been developed to effectively circumvent the limitations of 16S rDNA-based microbiota profiling (104). Techniques such as Smart‐seq, Smart‐seq2, MATQ‐seq (multiple annealing and dC‐tailing‐based quantitative single‐cell RNA‐seq), MARS‐seq (massively parallel single‐cell RNA‐sequencing), and CEL‐seq (Cell Expression by Linear amplification and sequencing) use fluorescence-activated cell sorting (FACS) for single-cell isolation (105–107). MARS‐seq and CEL‐seq amplify cDNA using an *in vitro* transcription method wherein the 5′ end of cDNA is connected to poly(A)/poly(C) to build common adaptors in the PCR reaction. Fluidigm C1, Smart‐seq2, MATQ‐seq, Drop-seq, and 10x Genomics use Moloney Murine Leukemia Virus (MMLV) reverse transcriptase to incorporate template‐switching oligos as adaptors for further PCR amplification. These methods also use unique molecular identifiers (UMI) to eliminate PCR amplification bias. Fluidigm C1 has two configurations: Fluidigm C1 96 scRNA-seq and Fluidigm C1 HT scRNA-seq, each tailored for different throughput requirements. This method captures and processes cells on an integrated microfluidic chip (IFC) with multiple capture sites, each capable of capturing a single cell. For library preparation, cDNAs were fragmented, barcoded, and amplified using the protocols recommended by Fluidigm. The libraries were then quantified and sequenced using high-throughput sequencing platforms such as Illumina HiSeq, NovaSeq and recently Nanopore has been used for the same (52, 108, 109). Despite encountering obstacles such as the diversity of cell walls, which are difficult to lyse, high abundance of rRNA, which makes it difficult to isolate non-polyadenylated mRNA (limited mRNA abundance), and mRNA instability, recent discoveries illustrate that single-cell techniques can capture bacterial sequences to a certain extent.

Well, understood and characterized intracellular survival strategies are limited to bacteria and a few parasites (at the moment) and are almost devoid for the fungal species. To understand how fungi manipulate their intracellular environment to ensure their persistence in mammalian hosts, it is necessary to investigate the specific mechanisms employed by these organisms. Fungi possess a rigid cell wall, which makes them less likely to be captured compared to bacteria and viruses (110). Additionally, they contain only 1-3 pg of total RNA per cell, which is 10 times less than that in mammalian cells, necessitating a specific RNA extraction procedure. Some methods are designed for single-cell sequencing of yeast organisms, such as Smart-seq, which uses zymolyase for efficient lysis of yeast cells within the droplets during single-cell droplet formation in the 10x Genomics Chromium Single Cell 3’ protocol (111). The authors validated these results using single-cell time-lapse microscopy. Yeast single-cell RNA sequencing (YscRNA-seq) is capable of detecting the expression of low-abundance noncoding RNAs and nearly half of the protein-coding genome in each cell. The cells were sorted using fluorescence-activated flow cytometry (FACS) in the 96-well plates, and full-length cDNA libraries were generated from biotinylated oligo(dT) and tagged libraries that were captured using streptavidin beads. In contrast, single-cell RNA barcoding and sequencing (SCRB-seq) specifically enriched 3’-end transcripts at the tagmentation stage after FACS sorting (112). However, these techniques have not yet been applied to human host cells; therefore, their diversity remains unknown. Several studies have demonstrated the diverse nature of the host response to antifungal infections through RNA-seq analysis of peripheral blood mononuclear cells (PBMCs) infected with various fungal species. For example, pathogens such as *Yersinia pestis* and *Candida albicans* invade and survive within macrophages as part of their life cycle (113, 114). These microorganisms cause a shift from pro-inflammatory to anti-inflammatory states and upregulate genes involved in the inflammasome activation, resulting in bimodality, and influencing infection outcomes in the healthy individuals. Moreover, fungal pathogens like *Cryptococcus neoformans, Histoplasma capsulatum, Candida glabrata, Candida albicans*, and *Aspergillus fumigatus* inhibit lysosomal fusion by altering intra-phagosomal pH, manipulating cytokine secretion, and inducing programmed cell death pathways such as pyroptosis and apoptosis. For instance, *Candida albicans* has been shown to trigger increased arginase activity upon sensing chitin, which leaves nitric oxide synthase (NOS) without a substrate and impairs macrophages’ ability to eliminate the fungus (115). This revelation not only broadens our understanding of microbial interactions within host-associated tissues but also presents a promising prospect for future therapeutic and disease applications. The advancement of integrated host-microbiome single-cell genomics and transcriptomics techniques has established a technological basis that may pave the way for personalized treatment strategies. By understanding complex host-microbiota interactions at the cellular level, these methods may provide insights into disease mechanisms and microbial pathogenesis, potentially resulting in targeted therapies and interventions.

## Knowledge gaps and future directions

The mechanisms underlying diverse infection outcomes due to host-microbe interactions in the presence of pathogens remain poorly understood. Infection outcomes are not solely defined by the primary pathogen infection abilities. Rather, pre-existing microbiota and host cellular factors play an important role. However, the existing tools for the identification of these intracellular microbes are still limited to obtain a better understanding of their existence and role inside definitive cell types. Hence, the development of new tools and computational pipelines is already forthcoming Several noteworthy research gaps have been identified. These lacunae serve as valuable points for future scientific inquiries. Foremost among these gaps is the pressing need to advance our holistic understanding of the intracellular microbiome. Presently, microbiome research predominantly focuses on the bacteriome, with an emphasis on anatomical regions such as the skin, oral cavity, nasal passages, and gastrointestinal tract. In stark contrast, there is a conspicuous dearth of attention paid to the intracellular diversity of microbiota. Profiling intracellular microbes, which encompass a diverse array of entities including bacteria, archaea, fungi, and viruses, presents serious challenges. These challenges stem from the scarcity of requisite technical and analytical resources, encompassing methodological approaches and computational pipelines. Moreover, genomic-based approaches and novel molecular-based technologies, including single-cell genomics, are becoming accessible, enabling the characterization of the transcriptome at the granular level. This greatly enhances our capacity to capture microbiome diversity and heterogeneity in a seemingly uniform cell population. However, thorough attempts to advance technologies used to analyze pathogens (such as bacteria, fungi, and viruses) accurately and efficiently at the single-cell level are critical. These limitations represent formidable barriers to researchers seeking to delve into the intricacies of the intracellular microbiome. scRNA-seq is an emerging technique to profile host and microbial transcriptomes. Unlike traditional approaches, scRNA-seq can simultaneously capture host and microbial transcriptomes, providing direct insight into host–microbe interactions. However, this part of scRNA-seq is still in its infancy, and it is only used to understand the transcript of either host or microbe. In light of these considerations, addressing these knowledge gaps and developing cutting-edge tools with respect to both experimental facets and computational pipelines to facilitate a comprehensive evaluation of the intracellular microbiome should assume a central position in future scientific investigations.

## Conclusion

The interactions between the intracellular microbiota and the host immune system are significant, as they play a critical role in shaping immune development and function. Additionally, they help maintain the symbiosis and integrity of the immune system. While techniques like microscopy, culturing, and DNA sequencing have been successful in environmental and human-associated microbiomes, identifying intracellular microbiomes remains challenging due to diverse cell wall structures and low mRNA abundances. However, single-cell genomics shows promise in capturing microbial sequences along with the host transcriptome, paving the way for joint host-microbiome transcriptome analysis. These advancements have revealed individual variability within the cell-type specific microbial communities, addressing their role in persistent infections and determining differential disease severity. Harnessing genomics strategies enables one to investigate the specific prevalence and function of microbes within the intricate environment of the host immune system. Intracellular microbes significantly impact disease outcomes by modulating cellular functions, yet our understanding of their role in disease severity is limited due to their unculturable nature. The implications of cell-to-cell variability (specifically immune cell types) towards internalizing specific microbes are unclear, yet significant in the background of the COVID-19 pandemic. Our recent lab work (DOI: 10.1016/j.isci.2023.108357) highlighted the non-canonical usage of Single Cell Transcriptomics to understand the microbial dynamics with immune cell types. This review highlights the intricate interactions of the intracellular microbiome, offering insights into predicting disease severity. This approach provides insight into the interactions, defense evasion mechanisms, and possible impact on varying disease outcomes. More innovations in the non-canonical genomic applications needs greater participation, integration, sharing and dissemination for public health benefits.

## Author contributions

RP: Conceptualization, Funding acquisition, Project administration, Resources, Supervision, Writing – review & editing. JS: Data curation, Formal analysis, Investigation, Resources, Visualization, Writing – original draft.
